# Reviewer acknowledgement 2015

**DOI:** 10.1186/s12934-016-0429-3

**Published:** 2016-02-17

**Authors:** Antonio Villaverde

**Affiliations:** Institut de Biotecnologia i de Biomedicina, Universitat Autònoma de Barcelona, Barcelona, Spain; Department de Genètica i de Microbiologia, Universitat Autònoma de Barcelona, Barcelona, Spain; CIBER de Bioingeniería, Biomateriales y Nanomedicina (CIBER-BBN), Zaragoza, Spain

*Microbial Cell Factories* would like to thank all reviewers who have contributed to the journal in 2015.

**Ibane Abasolo**

USA

**Cesar Aceves-Lara**

France

**Peter Temitope Adeboye**

Sweden

**Eduardo Agosin**

Chile

**Erika Agueiras**

Brazil

**Tatiana Aguiar**

Portugal

**Qazi Ahmed**

India

**Bilal Ahmed**

Pakistan

**Joong-Hoon Ahn**

South Korea

**Lianzhong Ai**

China

**Joan Albiol**

Italy

**Pietro Alifano**

Italy

**Ceren Alkim**

France

**Hal Alper**

USA

**Josef Altenbuchner**

Germany

**Isabel Amado**

Spain

**Diletta Ami**

Italy

**George Anderson**

USA

**Natalello Antonino**

Italy

**Jesús Aparicio**

Spain

**Joaquin Arino**

Spain

**Pankaj Arora**

South Korea

**Shota Atsumi**

USA

**Vasco Azevedo**

Brazil

**Loredana Baccigalupi**

Italy

**Dania Baccigapupi**

Italy

**Marcelo Baeza**

Chile

**Zhonghu Bai**

China

**Yuxiang Bai**

Netherlands

**Antonio Ballesteros**

Spain

**Mustafa Ozkan Baltaci**

Turkey

**Uttam Banerjee**

India

**François Baneyx**

USA

**Xiaoming Bao**

China

**Jie Bao**

China

**Ali Baradaran**

Australia

**Carlos Barreiro**

Spain

**Carl A. Batt**

USA

**Meike Baumgart**

Germany

**Karl Bayer**

Austria

**Erdal Bedir**

Turkey

**Harry Beller**

USA

**Stamatina Bellou**

Greece

**Kirsten Benjamin**

Germany

**Martina Bergant**

Slovenia

**Ales Berlec**

Slovenia

**Rita Bernhardt**

Germany

**Sanjoy K. Bhattacharya**

Canada

**Michele Bianchi**

Italy

**Mervyn Bibb**

UK

**Christophe Bignon**

France

**Roslyn Bill**

UK

**Josep Biosca**

Spain

**Virendra Bisaria**

USA

**Robert Bischof**

Austria

**Markus Bisschops**

Sweden

**Joerg Bohlmann**

Canada

**Eckhard Boles**

Germany

**Rajesh Reddy Bommareddy**

Germany

**Ursula Bond**

Ireland

**Anthony Borneman**

Australia

**Irina Borodina**

Denmark

**Lidia Boss**

Poland

**Daniel Bracewell**

UK

**Luca Brambilla**

Italy

**P. Bramley**

Japan

**Paola Branduardi**

Italy

**Stefania Brocca**

Italy

**Kirsi Bromann**

Finland

**José M. Bruno-Bárcena**

Spain

**Jochen Büchs**

Germany

**Bruno Bühler**

Germany

**Bruno Bühler**

Germany

**Matthias Bureik**

China

**Pietro Buzzini**

Italy

**María Ángeles Calvo**

Spain

**Carole Camarasa**

France

**Shawn Campagna**

USA

**Olivia Cano**

Spain

**Yujin Cao**

China

**Mingfeng Cao**

USA

**Pablo Carbonell**

UK

**Magnus Carlquist**

Sweden

**Mario Carrasco**

Chile

**Luis Caspeta**

Mexico

**Kathrin Castiglione**

Germany

**Tunc Catal**

Turkey

**M. Teresa Cesário**

Portugal

**M.V. Cespedes**

Spain

**Anju Chadha**

India

**Radka Chaloupkova**

Czech Republic

**Joe Chappell**

USA

**Jean-Marc Chatel**

France

**Guo-Qiang Chen**

China

**Tao Chen**

China

**Shouwen Chen**

China

**Yihua Chen**

China

**Xiaolin Chen**

China

**Min Chen**

China

**Shouwen Chen**

China

**Lei Chen**

China

**Kequan Chen**

China

**Qihe Chen**

China

**Haiqin Chen**

China

**Shaohua Chen**

Singapore

**Rachel Chen**

USA

**Fangyu Cheng**

China

**Michael Chikindas**

USA

**Sieo Chin Chin**

Malaysia

**Kwang Myung Cho**

South Korea

**Sol Choi**

South Korea

**Eui Sung Choi**

South Korea

**Anirban Roy Choudhury**

India

**Lukas Chrast**

Czech Republic

**M. Christodoulides**

UK

**Ju Chu**

China

**Maurizio Ciani**

Italy

**Donatella Cimini**

Italy

**Patrick Cirino**

USA

**Dennis Claessen**

Netherlands

**Muriel Cocaign-Bousquet**

France

**Fabian M. Commichau**

Germany

**Jonas Contiero**

Brazil

**José Luis Corchero**

Spain

**Don Cowan**

South Africa

**Nancy Da Silva**

USA

**André Ricardo Damasio**

Brazil

**Pascale Daran-Lapujade**

Netherlands

**Xavier Daura**

Spain

**Hazel Marie Davey**

UK

**Elisabetta De Alteriis**

Italy

**Jan-Willem De Gier**

Sweden

**Mayra de la Torre**

Mexico

**Ario de Marco**

Slovenia

**Steve delCardayre**

USA

**Frank Delvigne**

Belgium

**Mickael Desvaux**

France

**Raffaella Di Cagno**

Italy

**Ming-Zhu Ding**

China

**Tarek Dishisha**

Sweden

**Amanda Dodd**

South Africa

**Mike Domach**

USA

**Lucília Domingues**

Portugal

**Marina Donova**

Russia

**François Douillard**

Finland

**Martin Dragosits**

Austria

**Guocheng Du**

China

**Veeramuthu Duraipandiyan**

India

**Akio Ebihara**

Japan

**Anastassios Economou**

Greece

**Lothar Eggeling**

Germany

**Ahmed Elgendy**

Egypt

**Adelfo Escalante**

Mexico

**Baltasar Escriche**

Spain

**Jun Fan**

China

**Helisson Faoro**

Brazil

**Jorge Farias**

Chile

**I.M. Feavers**

UK

**Tamas Feher**

France

**Sun Fei**

China

**Jun Feng**

China

**Xueyang Feng**

USA

**Massimiliano Fenice**

Spain

**Ester Fernandez**

Spain

**Maria Fernandez**

Spain

**Pau Ferrer**

Spain

**Alastair Fleming**

Ireland

**Hooi Ling Foo**

Malaysia

**Marco Fraaije**

Netherlands

**Ricardo Franco-Duarte**

Portugal

**Paul Freemont**

UK

**Xiaozhi Fu**

China

**Pengcheng Fu**

USA

**Laurent Gal**

France

**Giuseppe Gallo**

Italy

**Amparo Gamero**

Spain

**Qiang Gao**

China

**Jose Luis Garcia**

Spain

**Faouzi Gargouri**

Tunisia

**Brigitte Gasser**

Austria

**D. Gayathri**

India

**Anli Geng**

Singapore

**George Georgiou**

USA

**Adrian Gerber**

Germany

**Sergio Giannattasio**

Italy

**Ryan T. Gill**

USA

**Benjamin Glick**

USA

**Ramon González**

Spain

**Sabina Górska**

Poland

**Guillermo Gosset**

Mexico

**Rita Grandori**

Italy

**Darren Greetham**

UK

**Reinhard Grisshammer**

USA

**Evamaria Gruchattka**

Germany

**Yingying Guo**

China

**Jian Guo**

China

**Martin Gustavsson**

Sweden

**Yoshihisa Hagihara**

Japan

**Mari Häkkinen**

Finland

**Dietmar Haltrich**

Austria

**Wenjun Han**

China

**Kiyotaka Hara**

Japan

**Hassan Hartman**

UK

**Rajni Hatti-Kaul**

Sweden

**Jun He**

China

**Michael Hecker**

Germany

**Stefan Heinl**

Austria

**Klaas Jan Hellingwerf**

Netherlands

**Michael Henson**

USA

**Yasuki Higashimura**

Japan

**Falk Hillmann**

Germany

**F. Hollmann**

Netherlands

**Sylvester Holt**

Belgium

**Kohsuke Honda**

Japan

**Bin Hong**

China

**Young-Soo Hong**

South Korea

**Walid A. Houry**

Canada

**Youjia Hu**

China

**Bo Hu**

USA

**He Huang**

China

**He Huang**

China

**Lixuan Huang**

USA

**Elton Hudson**

Sweden

**Iain Hunter**

UK

**Masayuki Inui**

Japan

**Alain Jacquet**

USA

**Rachit Jain**

USA

**Laura Jarboe**

USA

**Ki Jun Jeong**

South Korea

**Zueco Jesus**

Spain

**Zai-Si Ji**

China

**Xiaoqiang Jia**

China

**Rongrong Jiang**

Singapore

**Cheng Jin**

China

**Joachim Jose**

Germany

**Esther Julian**

Spain

**Héla Kallel**

Tunisia

**Sotirios C. Kampranis**

Greece

**Shin Kanamasa**

Japan

**Hirosuke Kanamoto**

Japan

**Hyun Ah Kang**

South Korea

**Martin Kangwa**

Germany

**Levente Karaffa**

Hungary

**Hamid Reza Karbalaei-Heidari**

Iran

**Kao Katy**

USA

**Yoshikazu Kawata**

Japan

**Oliver Kayser**

Germany

**Jay D. Keasling**

USA

**Navin Khanna**

India

**Kanchana Kildegaard**

Denmark

**Seung Wook Kim**

South Korea

**Beom Seok Kim**

South Korea

**Ok Bin Kim**

South Korea

**SungSoo Kim**

South Korea

**Seon-Won Kim**

South Korea

**Eung-Soo Kim**

South Korea

**Kohtaro Kirimura**

Japan

**Tobias Klement**

Germany

**Jan Dines Knudsen**

Italy

**Outi Koivistoinen**

Finland

**Akihiko Kondo**

Japan

**Jian-Qiang Kong**

China

**Robert Kourist**

Germany

**Florian Krainer**

Austria

**Vladimir Kren**

Czech Republic

**Manonmani Haravey Krishnan**

India

**Christian P. Kubicek**

Austria

**Galyna Kufryk**

USA

**Joosu Kuivanen**

Finland

**Mohit Kumar**

India

**Prasun Kumar**

India

**Antje Labes**

Germany

**Victor Ladero**

Spain

**Matic Lagisa**

Slovenia

**Göran Larson**

Sweden

**Christer Larsson**

Sweden

**Paola Laurienzo**

Italy

**Anburajan Lawrance**

India

**Sang Yup Lee**

South Korea

**Sung Kuk Lee**

South Korea

**Taek Soon Lee**

USA

**Han Lei**

China

**Eliana Lemos**

Brazil

**Rebecca Lennen**

Denmark

**Oliver Lenz**

Germany

**Serena Leone**

Italy

**Ana Ley**

Denmark

**Yin Li**

China

**Yue-zhong Li**

China

**Wenli Li**

China

**Ying Li**

China

**Ai-li Li**

USA

**Jiazhang Lian**

USA

**Jinping Lin**

China

**Zhanglin Lin**

China

**Jianping Lin**

China

**Geoff Lin-Cereghino**

USA

**Paloma Liras**

Spain

**Dehu Liu**

China

**Wan-Cang Liu**

China

**Xinxing Liu**

China

**Changhong Liu**

China

**Wen Liu**

China

**Jiayang Liu**

China

**Ziduo Liu**

China

**Weifeng Liu**

China

**Yi Liu**

China

**Tiangang Liu**

China

**Fang Liu**

USA

**Malgorzata Lobocka**

Poland

**Xuefeng Lu**

China

**Passaglia Luciane**

Brazil

**Gang Lui**

China

**Andriy Luzhetskyy**

Germany

**Hongwu Ma**

China

**Cuiqing Ma**

China

**Tien-Yang Ma**

Taiwan

**Robert Mach**

Austria

**Daniel Machado**

Portugal

**Astrid Rosa Mach-Aigner**

Austria

**John MacMillan**

USA

**Knut Madden**

USA

**Antonios Makris**

Greece

**Uwe Mamat**

Germany

**Pin-Ching Maness**

USA

**Isabel Manso Cobos**

Spain

**Takashi Maoka**

Japan

**Costas Maranas**

USA

**Jan Marienhagen**

Germany

**Monica Marin**

Uruguay

**Christopher Marquis**

Australia

**Juan F. Martin**

Spain

**Manuele Martinelli**

Italy

**Alfredo Martinez**

Mexico

**Miguel A. Martinez**

Spain

**Hans Marx**

Austria

**Malihe Masomian**

Malaysia

**Shinji Masuda**

Japan

**Geir Mathiesen**

Norway

**Inga Matijosyte**

Lithuania

**Diethard Mattanovich**

Austria

**Ana Mendes-Ferreira**

Portugal

**P. Messi**

Italy

**Andreas Meyerhans**

USA

**Bashir Mienda**

Malaysia

**Igor Mierau**

Netherlands

**Ali Mohagheghi**

USA

**Venkata Mohan**

India

**Tapan Kumar Mohanta**

South Korea

**Pradeep Kumar Das Mohapatra**

India

**Dominik Mojzita**

Finland

**Carlos Molina-Santiago**

Spain

**Veronique Monnet**

France

**Suzy Moody**

UK

**Patrice Moreau**

France

**Tomotoake Morita**

Japan

**Uffe Mortensen**

Denmark

**Jean-Roch Mouret**

France

**Arturo Muga**

Spain

**Howbeer Muhamadali**

UK

**Serge Muyldermans**

Belgium

**Todd Naumann**

USA

**Gennadi Ivanovich Naumov**

Russia

**Juana Maria Navarro Llorens**

Spain

**Rodrigo Navia**

Chile

**Hadi Nazem-Bokaee**

USA

**Juan Alejandro Negrete Virgen**

USA

**Peter Neubauer**

Germany

**T.T. Nguyen**

Vietnam

**Jens Nielsen**

Denmark

**Alex Nielsen**

Denmark

**Hiroshi Nikaido**

USA

**Pablo Ivan Nikel**

Spain

**Stephan Noack**

Germany

**Joakim Norbeck**

Sweden

**Berl Oakley**

USA

**Kozo Ochi**

Japan

**Zach Oestreicher**

USA

**Naotake Ogasawara**

Japan

**Kenji Okano**

Japan

**Maria Oliveira**

Brazil

**Giuseppe Olivieri**

Netherlands

**Pawel Olszewski**

Poland

**Margarita Orejas**

Spain

**Toomas Paalme**

Estonia

**Claudio Palleschi**

Italy

**Laura Palomares**

Mexico

**Jose M. Palomo**

Spain

**Amulya Panda**

India

**Maria Papagianni**

Greece

**Seraphim Papanikolaou**

Greece

**Enoch Park**

Japan

**Si Jae Park**

South Korea

**Cheon-Seok Park**

South Korea

**Sunghoon Park**

South Korea

**Jin-Byung Park**

South Korea

**Ermenegilda Parrilli**

Italy

**Luís Passarinha**

Portugal

**Raosahelb Patil**

Germany

**Fabio Pedrosa**

Brazil

**Roberto Pérez-Torrado**

Spain

**Mireia Pesarrodona**

Spain

**Hrvoje Petkovic**

Slovenia

**Kaloyan Petrov**

Bulgaria

**Uroš Petrovič**

Slovenia

**Harald Pichler**

Austria

**Jeff Piotrowski**

USA

**Karen Polizzi**

UK

**David Pollard**

UK

**Isabelle Poquet**

France

**Danilo Porro**

Italy

**Bruno Pot**

France

**Qingsheng Qi**

China

**Amparo Querol**

Spain

**Amparo Querol**

Spain

**Ayyalusamy Ramamoorthy**

USA

**GanQiao Ran**

China

**Zhiming Rao**

China

**Rajko Reljic**

UK

**Yilin Ren**

China

**Rodrigo Resende**

Brazil

**Jose L. Revuelta**

Spain

**Moez Rhimi**

France

**Juan Rico Molins**

France

**Ursula Rinas**

Germany

**Ligia Rodrigues**

Portugal

**Manuel Rodríguez-Concepción**

Spain

**Nerea Roher**

Spain

**Ulrika Rova**

Sweden

**Utpal Roy**

India

**Lloyd Ruddock**

Finland

**Karl Rumbold**

South Africa

**Stefan Ruyters**

Belgium

**Margherita Sacco**

Italy

**Kobra Saeidi**

Iran

**Katsuichi Saito**

Japan

**Georgina Sandoval**

Mexico

**Ramón Santamaría**

Spain

**Javier Santos-Aberturas**

Spain

**Dimitirs Sarris**

Greece

**Michael Sauer**

Austria

**Anett Schallmey**

Germany

**Wilfried Schwab**

Germany

**Thomas Schweder**

Germany

**Paul Schweiger**

USA

**Francesco Secundo**

Italy

**Bernhard Seiboth**

Austria

**Richard Selvam**

India

**Manjit K. Selwal**

India

**Juzheng Sheng**

China

**Elizabeth Shephard**

UK

**Junling Shi**

China

**Joseph Shiloach**

USA

**Bun-Ichi Shimizu**

Japan

**Hiroshi Shimizu**

Japan

**Andriy Sibirny**

Ukraine

**Verena Siewers**

Sweden

**Giuliano Siligardi**

UK

**Edson Silva**

Brazil

**Steven Singer**

USA

**O. Singh**

USA

**Arne Skerra**

Germany

**Rimantas Slibinskas**

Lithuania

**Christina Smolke**

USA

**Jae Kyung Sohng**

South Korea

**Cunjiang Song**

China

**Hans Peter Sørensen**

Denmark

**Oliver Spadiut**

Austria

**Terrance A. Stadheim**

USA

**Matthias Steiger**

Austria

**Lisa Stein**

Canada

**Marc Stevens**

USA

**Gisela Storz**

USA

**Haifeng Su**

China

**Minetaka Sugiyama**

Japan

**Ryan Summers**

USA

**Fei Sun**

China

**Zhongke Sun**

China

**Per Sunnerhagen**

Sweden

**John B. Sutherland**

USA

**Shang-Tian Yang**

USA

**T Tajima**

Japan

**Kaoru Takegawa**

Japan

**Furong Tan**

China

**Yinjie Tang**

USA

**Andreas Tauch**

Germany

**Hilal Taymaz-Nikerel**

Belgium

**Eleni Theodosiou**

Germany

**Johan Thevelein**

Belgium

**Muriel Thomas**

France

**lya Tolstorukov**

USA

**Bryan Tracy**

USA

**Armen Trchounian**

Armenia

**Cong Trinh**

USA

**Sucheta Tripathy**

India

**Maria Luisa Tutino**

Italy

**Vlada Urlacher**

Germany

**Norma Adriana Valdez-Cruz**

Mexico

**Francisco Valero**

Spain

**Antonio Valle**

Spain

**Stephen Van Dien**

USA

**Richard van Kranenburg**

Netherlands

**Willem H. van Zyl**

Denmark

**Mario Varcamonti**

Italy

**S. Venkata Mohan**

India

**Michelle Visser**

USA

**Thomas Vogl**

Australia

**Bjorn Voldborg**

Denmark

**Aljoscha Wahl**

Netherlands

**Xia Wan**

China

**Lu Wang**

China

**Qinhong Wang**

China

**Yujiong Wang**

China

**Jian Wang**

China

**Zhiwen Wang**

China

**Depei Wang**

China

**Jin Wang**

China

**Ganggang Wang**

China

**Bo Wang**

USA

**Grzegorz Wegrzyn**

Poland

**Alicja Wegrzyn**

Poland

**Gongyuan Wei**

China

**Jianping Wen**

China

**Volker F. Wendisch**

Germany

**Cui Wenjing**

China

**Thomas West**

USA

**Ian Wheeldon**

USA

**Barrie Wilkinson**

UK

**Paul Wilmes**

Luxembourg

**Margit Winkler**

Austria

**Keehoon Won**

South Korea

**David W. Wood**

USA

**Mhairi Workman**

Denmark

**Hui Wu**

China

**Zhenqiang Wu**

China

**Pengfei Wu**

China

**Huanzhang Xia**

China

**Fei Xiao**

China

**Sidong Xiong**

China

**Zhaoxian Xu**

China

**Gang-Ming Xu**

China

**Yi-Gang Xu**

China

**Ping Xu**

China

**Guoqiang Xu**

China

**Montarop Yamabhai**

Thailand

**Yajun Yan**

China

**Chuan Mei Yeh**

Taiwan

**Jin Yin**

China

**Yeo Joon Yoon**

South Korea

**Huimin Yu**

China

**Bo Yu**

China

**Jianping Yu**

USA

**Zehra Nur Yuksekdag**

Turkey

**Jurgen Zanghellini**

Austria

**Xianhai Zeng**

China

**Jixun Zhan**

USA

**Dawei Zhang**

China

**X. Zhang**

China

**Yifeng Zhang**

China

**Guoqiang Zhang**

China

**Jianan Zhang**

China

**Yiming Zhang**

Sweden

**Wenjun Zhang**

USA

**Han Zhao**

China

**Guang Zhao**

China

**Zhou Zhemin**

China

**Huajun Zheng**

China

**Daoqiong Zheng**

China

**Xiangshan Zhou**

China

**Jingwen Zhou**

China

**Zhi Zhou**

USA

**Ping Zhu**

China

**Emanuele Zonaro**

Italy

